# Recent Updates and Advancements in the Diagnosis and Management of Plasma Cell Dyscrasias

**DOI:** 10.7759/cureus.101535

**Published:** 2026-01-14

**Authors:** Anand Sunil, Ahnaf Uddin Ahmed, Ahmed Elkhider Ali Musa, Arzoo Dar, Nafrin Kormath, Sai Lahari Sangaraju, Nejma Azeez, Bashir Imam, Vinay Dontul, Sweta Singh, Hussein Attia Hussein Mahmoud, Manju Rai

**Affiliations:** 1 Internal Medicine, Manchester University NHS Foundation Trust, Manchester, GBR; 2 Internal Medicine, Trinity Biomedical Sciences Institute, Dublin, IRL; 3 Family Medicine, University of Medical Sciences and Technology, Khartoum, SDN; 4 Medicine, Hull York Medical School, York, GBR; 5 Internal Medicine, Muslim Education Society (MES) Medical College Hospital, Perinthalmanna, IND; 6 Internal Medicine, People’s Education Society (PES) Institute of Medical Science and Research, Kuppam, IND; 7 Internal Medicine, East Sussex Health Care NHS Trust, Eastbourne District General Hospital, East Sussex, GBR; 8 Pediatric, University of Pittsburgh Medical Center, Coudersport, USA; 9 Internal Medicine, Apollo Institute of Medical Sciences and Research, Hyderabad, IND; 10 Internal Medicine, Maharashtra University of Health Sciences, Nashik, IND; 11 Diagnostic Radiology, Heliopolis Hospital, Cairo, EGY; 12 Biotechnology, Shri Venkateshwara University, Uttar Pradesh, IND

**Keywords:** artificial intelligence, bispecific antibodies, car-t, genomics, immunotherapy, mass spectrometry, minimal residual disease, multiple myeloma, plasma cell dyscrasias, supportive care

## Abstract

Plasma cell dyscrasias (PCDs) encompass a heterogeneous group of disorders ranging from asymptomatic precursor states such as monoclonal gammopathy of undetermined significance (MGUS) and smoldering multiple myeloma (SMM) to overt multiple myeloma (MM), primary plasma cell leukemia, and systemic amyloidosis. This review is based on a structured search of PubMed, Scopus, Cochrane Library, and Web of Science for literature published between 2015 and 2025, supplemented by reference screening and major international myeloma guidelines to ensure comprehensive coverage of recent advancements. Recent years have witnessed remarkable progress in understanding disease biology, refining diagnostic tools, and expanding therapeutic strategies. Advances in genomics and cytogenetics have deepened insight into clonal evolution and prognostic markers, while next-generation flow cytometry, mass spectrometry, and high-sensitivity serum free light chain assays are revolutionizing disease detection and minimal residual disease monitoring. Parallel improvements in imaging, including whole-body MRI and novel PET tracers, enhance accuracy in disease assessment, while artificial intelligence-driven models hold promise for predictive analytics and personalized care. Therapeutically, immunotherapies including monoclonal antibodies, bispecific antibodies, and chimeric antigen receptor T (CAR-T) cell therapies have transitioned from salvage settings to frontline use, providing deeper and more durable responses. Risk-adapted approaches, improved transplant strategies, and novel maintenance regimens are reshaping the standard of care. Advances also extend to the management of non-myeloma PCDs, supportive care, and survivorship, underscoring the importance of patient-centered approaches. Despite these gains, challenges persist in overcoming therapy resistance, managing costs, and ensuring equitable global access to novel treatments. Looking forward, integration of multi-omics with artificial intelligence, expansion of collaborative clinical trials, and strategies balancing cure vs. disease control will be pivotal in optimizing outcomes. This review synthesizes current evidence and highlights emerging directions shaping the evolving landscape of PCD management.

## Introduction and background

Over the past decade, the field of plasma cell dyscrasias (PCDs) has undergone several fundamental shifts that warrant an updated, integrative review. Diagnostic paradigms have expanded beyond conventional electrophoretic techniques to include mass spectrometry (MS)-based assays, next-generation flow (NGF) cytometry and sequencing for minimal residual disease (MRD) assessment, and increasingly sensitive whole-body imaging. These advances now allow detection of disease at a much lower burden, earlier identification of relapse, and more precise response evaluation than was previously possible in routine practice.

At the same time, therapeutic strategies have evolved rapidly. The introduction of immunotherapy-based approaches, including anti-CD38 monoclonal antibodies, bispecific T-cell engagers, and chimeric antigen receptor T (CAR-T) cell therapies, has reshaped treatment algorithms, delivering deeper and more durable responses across multiple disease stages. Increasing use of risk-adapted and response-guided strategies, particularly those incorporating MRD status, reflects a broader shift toward personalized care rather than uniform treatment pathways.

For clinicians, these developments translate into meaningful changes in everyday decision making: earlier confirmation of disease in diagnostically challenging cases, more accurate assessment of treatment depth, and greater flexibility in tailoring therapy intensity based on biological risk and patient-specific factors.

PCDs comprise a spectrum of disorders arising from clonal proliferation of plasma cells, ranging from asymptomatic precursor states such as monoclonal gammopathy of undetermined significance (MGUS) and smoldering multiple myeloma (SMM), through overt multiple myeloma (MM), to aggressive and rare variants including primary plasma cell leukemia (PCL), non-secretory myeloma, and immunoglobulin isotype-defined subtypes (e.g., IgD, IgE) [1,2]. Rather than being isolated entities, these conditions are interconnected by shared biological features (e.g., genomic instability, monoclonal immunoglobulin production) but vary greatly in risk of progression, clinical presentation, and therapeutic needs. 

In 2025, PCDs remain a major global health challenge. The global incidence and burden of MM continue to rise: a systematic analysis of the Global Burden of Disease Study (1990-2021) shows increasing age-standardized incidence, mortality, and disability-adjusted life years (DALYs), particularly in regions with high sociodemographic indices, underscoring both demographic shifts and unequal access to diagnostics and care [3]. Clinically, PCDs pose challenges in early detection (especially of rare subtypes or non-secretory disease), heterogeneity in response to treatment, and management of organ damage (bone disease, renal involvement, etc.). Economically, the cost burden is substantial: both novel diagnostics and therapies contribute to high direct medical costs, while hospitalizations, treatment of complications and relapses, and supportive care drive further expenditure [4,5].

Over the past decade, several paradigm shifts have transformed the diagnosis and management of PCDs. Genomic profiling has revealed increasingly better characterization of driver mutations, cytogenetic aberrations, and clonal evolution underlying not just overt MM but even in precursor states, refining risk stratification and opening the door to targetable vulnerabilities [6]. Imaging modalities have evolved: whole-body 18F-FDG PET/CT, hybrid PET/MRI, and the use of functional MRI (e.g., diffusion-weighted imaging) now allow detection of bone and extramedullary disease (EMD) with greater sensitivity, earlier than traditional skeletal surveys, and are increasingly used for response assessment. Novel tracers (e.g., 68Ga-Pentixafor) are under study for detecting MRD non-invasively [7,8]. Therapeutically, immunotherapies such as bispecific antibodies (BsAbs), CAR-T cell therapies, and monoclonal antibodies (e.g., anti-CD38 agents) have moved from salvage settings into earlier lines of therapy, delivering deeper, more durable responses and reshaping the treatment landscape [9,10].

The objective of this review is to integrate these cutting-edge diagnostic modalities and therapeutic strategies, genomics, imaging, and immunotherapy into a future-facing clinical roadmap. We aim to delineate how recent advances can inform earlier detection, individualized risk stratification, optimal therapy selection (including for rare and high-risk PCD subtypes), and sustainable healthcare delivery. Through this narrative synthesis, we hope to offer clinicians and researchers guidance on how the diagnosis and management of PCDs are likely to evolve in the coming years, and what practice changes may be most impactful in improving patient outcomes.

## Review

Methodology

**Figure 1:**
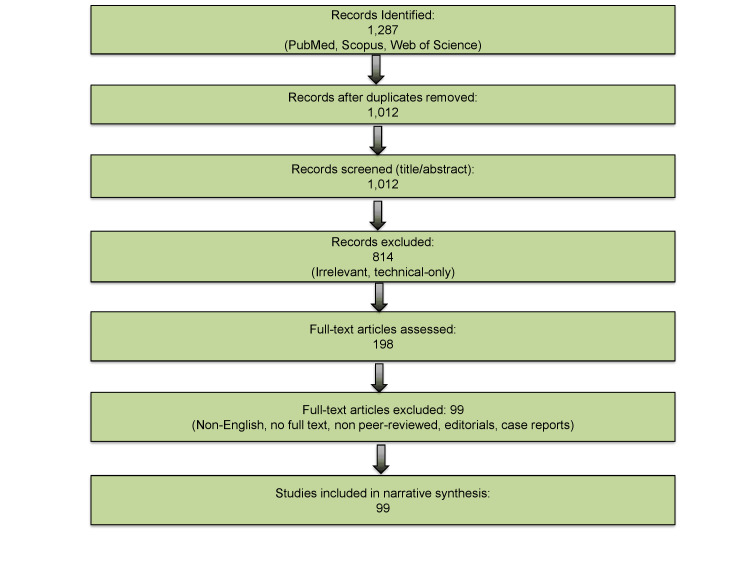
Overview of the steps involved in identifying and selecting studies included in the narrative review

This narrative review was conducted to synthesize recent literature and emerging evidence related to the diagnosis and management of PCDs. A comprehensive search of PubMed, Scopus, and Google Scholar was performed for articles published between 2015 and 2025, using key terms such as “plasma cell dyscrasias,” “multiple myeloma,” “mass spectrometry,” “minimal residual disease,” “CAR-T,” “bispecific antibodies,” and “artificial intelligence.” Additional references were identified from bibliographies of relevant studies and consensus guidelines from the International Myeloma Working Group (IMWG). Only peer-reviewed English-language articles, systematic reviews, randomized clinical trials, and major observational studies were included. Editorials, commentaries, conference abstracts without full peer-reviewed publication, single case reports, and non-English-language articles were excluded from the final narrative synthesis. Data were critically analyzed to identify diagnostic innovations, therapeutic advances, and evolving management trends. The findings were qualitatively synthesized and structured thematically to provide a comprehensive and updated overview. No new patient data were collected, and ethical approval was not required. The study selection process is summarized in Figure 1.

Revolution in diagnostics: beyond conventional electrophoresis

Recent advances in diagnostic technologies, including MS, ultrasensitive serum free light chain (sFLC) assays, NGF cytometry, and advanced imaging, enable earlier, more sensitive, and more precise detection of PCDs, particularly in low-burden, oligosecretory, or non-secretory disease, and are reshaping standards for disease assessment and monitoring. In routine practice, patients may present with clinical or radiologic features suggestive of active or relapsing disease despite negative or equivocal results on conventional serum protein electrophoresis (SPEP) or immunofixation. In such scenarios, advanced diagnostic modalities can facilitate earlier confirmation of residual or recurrent disease, improve risk stratification, and guide timely treatment decisions that would otherwise be delayed using traditional assays alone.

Limitations of Traditional Tools (SPEP, Urine Protein Electrophoresis (UPEP), and Immunofixation)

**Figure 2:**
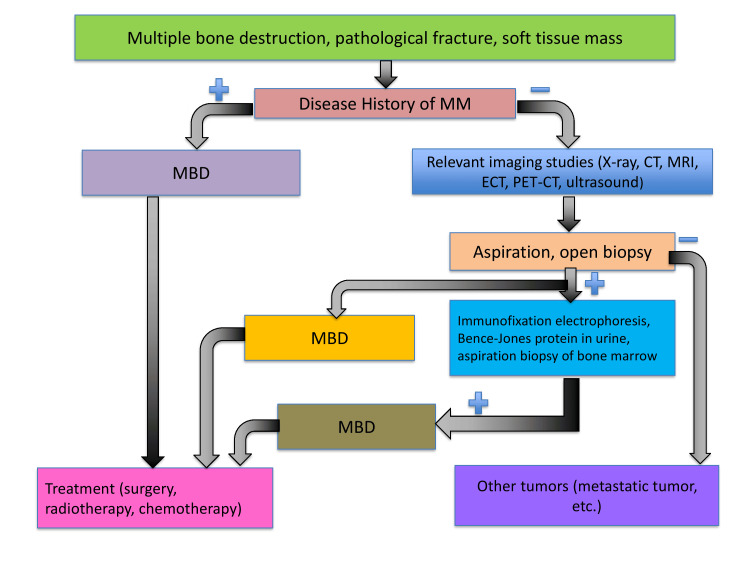
Diagnostic workflow for multiple bone destruction (MBD) in suspected multiple myeloma (MM) This flowchart illustrates the stepwise approach to evaluating patients presenting with multiple bone lesions, pathological fractures, or soft tissue masses. In patients with a prior history of MM, MBD is presumed and treated accordingly. In patients without known MM, relevant imaging studies (X-ray, CT, MRI, ECT, PET-CT, ultrasound) are performed. If lesions are suspicious, aspiration or open biopsy is undertaken, followed by immunofixation electrophoresis, detection of Bence-Jones protein in urine, and bone marrow aspiration biopsy. Based on findings, MBD related to MM is distinguished from other metastatic tumors, guiding further treatment strategies including surgery, radiotherapy, or chemotherapy [7-8]. Image credit: Ahmed Elkhider Ali Musa

SPEP, UPEP, and immunofixation electrophoresis (IFE) have long formed the diagnostic backbone for PCDs. However, they suffer from several deficiencies. SPEP cannot reliably detect low-level monoclonal proteins (<0.5 g/dL), struggles with co-migration of proteins, and gives little qualitative detail beyond the large “M-spikes.” UPEP is cumbersome (24-hour collections), has poor patient compliance, and lower sensitivity. IFE, while qualitative, has low throughput, subjective interpretation differences across labs, and interference by therapeutic monoclonal antibodies [3,8,10]. These tools also underperform in oligosecretory, nonsecretory, or light chain only disease; in monitoring MRD, conventional electrophoretic techniques often cannot detect very low disease burdens. The diagnostic evaluation of patients presenting with multiple bone destruction, fractures, or soft tissue masses often requires an integrated approach combining imaging, histopathology, and laboratory studies to distinguish MM from other tumors (Figure 2).

MS-Based Assays Replacing Immunofixation

MS methods are now showing evidence that they can supplant IFE in many settings. The MASS-FIX assay (a matrix-assisted laser desorption/ionization (MALDI-TOF)-based intact light chain MS assay), when deployed in large cohorts (e.g., ~19,500 patients), proved more sensitive than IFE in confirming SPEP abnormalities, detecting small monoclonal proteins that SPEP could barely quantify, and showing greater automation and lower repeat rates. In that study, MASS-FIX detected M-proteins in samples SPEP identified as suspicious but not quantifiable, and in samples with abnormal FLC ratios, it outperformed or matched IFE [11]. The IMWG Mass Spectrometry Committee has, in its report, endorsed immune-enrichment coupled with MALDI-TOF MS (or LC-MS) as clinically equivalent or superior to IFE for diagnosis/monitoring of monoclonal proteins, differentiating therapeutic antibodies from disease, and improving detection of risk features such as light chain glycosylation [12]. Personalized MS-peptide/clonotypic assays such as “EasyM” have pushed detection limits very low (on the order of 0.0005-0.05 g/L for some peptides), and offer quantitative tracking in addition to qualitative presence/absence [13]. While MS has challenges - cost, equipment, and a need for standardization - the evidence suggests it is ready to replace or run in parallel with IFE in many diagnostic workflows.

Several of these studies represent practice-changing diagnostic advances, particularly large cohort and consensus reports demonstrating the superiority of MS over immunofixation for detecting low-level monoclonal proteins and distinguishing therapeutic antibodies from disease-related clones. In contrast, personalized peptide-based assays and novel blood-based MRD approaches currently constitute emerging evidence, with promising sensitivity but more limited real-world validation.

Advances in sFLC Assay: Ultrasensitive Quantification

Parallel to MS advances, the sFLC assay has seen refinements in reference intervals, prognostic power, and analytic performance. A large retrospective study (≈95,000 SPEP/IFE + sFLC specimens over a decade) demonstrated that the widely used manufacturer-recommended κ/λ ratio (0.26-1.65) led to a high false-positive rate among monoclonal-negative individuals; using real-world data, revised reference intervals (e.g., 0.67-2.13 for the Optilite platform) reduced false positives substantially without loss of sensitivity [14]. A Danish population study of over 13,000 patients compared SPEP + UPEP vs. SPEP + sFLC ratio in general practice; combined with SPEP, the sFLC ratio nearly matched UPEP in sensitivity, and modifying cut-offs reduced false positives [15]. Also, in MM cohorts treated with novel agents, serial sFLC measurements have shown earlier detection of response/progression than electrophoretic methods; sFLC changes often preceded urine M-protein or SPEP changes by >1 month [16-17]. These improvements push us toward ultrasensitive quantification, with better prognostication, more timely detection of relapse (including sFLC-only relapse), and enhanced ability to define stringent complete response (sCR).

NGF Cytometry for MRD

High-sensitivity MRD assessment using NGF cytometry, next-generation sequencing, advanced imaging, and emerging blood-based approaches provides a powerful prognostic tool that increasingly informs response assessment, treatment duration, and risk-adapted therapeutic strategies in PCDs. In patients achieving a conventional complete response, residual disease may persist at levels undetectable by standard methods, contributing to relapse risk. MRD assessment allows clinicians to identify patients with ongoing subclinical disease who may benefit from treatment intensification or prolonged maintenance, while also supporting de-escalation strategies in deeply responding, MRD-negative patients to reduce cumulative toxicity and preserve quality of life (QoL).

Real-world experience from a large reference laboratory that reflexed lower-sensitivity screening assays to high-sensitivity flow cytometry analyzed >13,000 bone marrow specimens and demonstrated that many samples negative by routine screening were MRD-positive when assessed with next-generation techniques, underscoring the clinical impact of assay sensitivity in routine practice [18]. NGF, standardized by the EuroFlow consortium, employs harmonized antibody panels and acquisition/analysis pipelines to reproducibly reach sensitivities of ~10⁻⁵-10⁻⁶ and has been widely adopted in trials and reference centers [19]. Head-to-head prospective data from contemporary trials (e.g., the FORTE program) show strong concordance between NGF and next-generation sequencing (NGS) at comparable cutoffs and confirm that sustained MRD negativity by either modality predicts markedly improved progression-free and overall survival [20]. Practically, NGF offers rapid turnaround, no requirement for a diagnostic DNA sample, and the ability to phenotype residual malignant plasma cells, though preanalytical variables (aspirate hemodilution, panel standardization) remain critical to achieving true 10⁻⁶ performance.

Hence, large prospective trials and standardized EuroFlow-based studies constitute landmark evidence establishing MRD negativity as a robust prognostic marker, while ongoing investigations are evaluating how MRD-directed strategies may be optimally integrated into routine, response-adapted clinical decision making.

Imaging Breakthroughs: Whole-Body MRI, PET/CT With Novel Tracers

International imaging recommendations now endorse sensitive whole-body techniques for staging and response assessment, recognizing that marrow heterogeneity and EMD can be missed by marrow-only testing [21]. Molecular imaging advances complement these recommendations: CXCR4-targeted 68Ga-Pentixafor PET/CT has demonstrated complementary lesion detection compared with 18F-FDG in multiple series and may identify biologically aggressive, CXCR4-positive disease amenable to theranostic approaches [22]. Standardization efforts such as MY-RADS for whole-body MRI (diffusion-weighted sequences) improve acquisition and reporting consistency, enabling WB-MRI to detect marrow involvement with high sensitivity and to serve as a noninvasive adjunct for treatment response and MRD-directed strategies [23].

Artificial Intelligence (AI) and Machine Learning in Early Diagnosis and Prognostic Prediction

AI and machine-learning tools are increasingly applied to automate and quantify imaging (e.g., volumetric metabolic tumor volume, total lesion glycolysis), flow cytometry gating, and integrative prognostic modeling. Automated AI-based volumetric segmentation of FDG PET/CT correlates with marrow infiltration and independently predicts survival outcomes, illustrating reproducible, scalable imaging biomarkers for risk stratification [24]. Larger transformer-based models that combine clinical, laboratory, and imaging data have improved prediction of progression-free and overall survival and adverse events compared with traditional staging systems, pointing toward individualized, data-driven prognostic frameworks for clinical decision making [25]. Together, NGF/NGS, advanced imaging, and AI provide a spatially and molecularly informed diagnostic ecosystem that enables MRD-driven, precision care pathways. Table 1 summarizes the emerging diagnostic modalities in PCDs, highlighting their key features, advantages, limitations, and clinical applications.

**Table 1:** Emerging diagnostic modalities in plasma cell dyscrasias Abbreviations: 68Ga, gallium 68; DNA, deoxyribonucleic acid; EasyM, Easy Monitoring; IFE, immunofixation electrophoresis; MASS-FIX, mass spectrometry-based free light chain assay; MRI, magnetic resonance imaging; MRD, minimal residual disease; NGF, next-generation flow cytometry; NGS, next-generation sequencing; OS, overall survival; PET, positron emission tomography; PFS, progression-free survival

Modality	Key Features	Advantages	Limitations	Clinical Application	Reference
Mass Spectrometry (MASS-FIX, EasyM)	Detects low-level M-proteins	High sensitivity, automated	Cost, standardization issues	Replacing IFE, MRD detection	[11-13]
Serum Free Light Chain Assay (Ultrasensitive)	κ/λ ratio, prognostic	Early relapse detection, serial monitoring	Platform variability	Prognostication, response assessment	[14-17]
NGF	Sensitivity 10⁻⁵-10⁻⁶	Standardized, rapid, phenotyping	Requires marrow sample, dilution risk	MRD assessment	[18-20]
NGS	Detects clonal DNA	High precision, sustained MRD tracking	Needs baseline sample, cost	Trials, MRD	[20]
Imaging (PET/MRI, 68Ga-Pentixafor PET)	Functional + molecular info	Detect extramedullary and marrow disease	Availability, cost	Staging, MRD, theranostics	[21-23]
AI and Machine Learning	Integrates multi-omics and imaging	Predicts PFS/OS, decision support	Requires large datasets, validation	Personalized medicine	[24-25]

Redefining clinical spectrum: novel subtypes and overlaps

The clinical spectrum of PCDs continues to expand, with recognition and characterization of both very rare subtypes and overlapping syndromes that blur traditional boundaries. These novel entities and overlaps have implications for diagnosis, prognostication, and therapy.

Non-traditional Entities: IgE/IgD Myeloma, Biclonal Gammopathies

IgE myeloma is among the rarest isotypes (<0.1% of all MM cases), typically associated with aggressive biology. A recent genomic and immune repertoire profiling study of IgE κ MM detailed a hypermutated phenotype and heightened T cell reactivity, shedding light on its molecular distinctiveness and potential immunologic vulnerabilities [26]. Case reports also document that IgE MM can be underdiagnosed when standard immunofixation and electrophoresis miss weak bands, and that early use of novel agents combined with stem cell transplantation may improve outcomes in select cases [27]. Biclonal gammopathies, the occurrence of two distinct monoclonal immunoglobulins, are uncommon (≈1%), but series from tertiary centers (e.g., India) show presentation, heavy‐light chain combinations (e.g., IgG/IgA) similar to monoclonal disease, and comparable response to standard therapies when both clones are addressed [28].

PCDs Intersecting With Autoimmune and Paraneoplastic Syndromes

There is growing evidence for interactions between autoimmune processes and PCDs. A recent comprehensive review analyzed correlations among autoimmune diseases and conditions such as MGUS and MM; some autoimmune disorders are statistically associated with increased risk of MGUS/MM, potentially via chronic immune stimulation, cytokine milieu, or shared genetic susceptibility [29]. Clinical cases where monoclonal gammopathy (including MGUS) leads to organ damage via autoantibody‐mediated mechanisms (e.g., nephropathy, neuropathy) emphasize that “undetermined significance” is not always benign [30].

The Expanding Landscape of Amyloidosis and POEMS With New Classification Tools

The International Society of Amyloidosis (ISA) released updated nomenclature in 2024, identifying 42 distinct human amyloid fibril proteins, 19 of which cause systemic forms, refining the classification of amyloid diseases and enabling better diagnostic precision for more rare fibril types [31]. POEMS (Polyneuropathy, Organomegaly, Endocrinopathy, Monoclonal plasma cell disorder, Skin changes) syndrome remains rare, but recent work has delineated the “capillary‐leak phenotype” as a distinct clinical cause of morbidity and mortality in POEMS, suggesting that disease phenotyping beyond the classic criteria merits integration into prognostic stratification [32].

Concept of “Oligoclonal Reconstitution” Post-therapy

Oligoclonal reconstitution refers to the emergence of immunoglobulin bands distinct from the diagnostic clone after therapy (especially after autologous stem cell transplant or induction with novel agents). Several studies show that this phenomenon (sometimes called oligoclonal bands or immune reconstitution) is associated with deeper responses, higher rates of MRD negativity, and improved progression‐free survival [33,34]. For example, in a cohort of 156 patients undergoing ASCT, those with oligoclonal band formation had significantly longer PFS and OS compared to those without [33]. While not yet uniformly incorporated into response criteria, oligoclonality represents a marker of immune restoration and possibly a favorable prognosis, though it complicates sCR definitions due to effects on FLC ratios.

Management frontiers: tailoring therapy in the precision era

Immunotherapy-based strategies, including anti-CD38 monoclonal antibodies, bispecific T-cell engagers, and CAR-T cell therapies, have transformed the therapeutic landscape of PCDs by delivering deeper and more durable responses, expanding effective options across disease stages, and enabling increasingly personalized, risk-adapted treatment approaches. For patients with relapsed or refractory disease, particularly those with high-risk cytogenetics or early relapse after standard regimens, immunotherapies now offer clinically meaningful responses where conventional therapies often fail. In real-world settings, clinicians must integrate these agents thoughtfully, balancing depth of response against toxicity, infection risk, access, and cost, while considering earlier use of immunotherapy in selected high-risk or MRD-positive patients.

Induction and Early Therapy

The therapeutic backbone for newly diagnosed multiple myeloma (NDMM) has shifted decisively toward deeper, immunologically-enabled induction regimens that combine next-generation proteasome inhibition (e.g., carfilzomib), modern IMiDs, and anti-CD38 monoclonal antibodies [35-37]. Carfilzomib-based regimens (KRd) showed potent activity and higher complete response rates compared with older PI backbones in several studies, but clinicians must weigh efficacy against demonstrated risks (notably cardiovascular toxicity) and tailor choice on comorbidity profiles [35]. Anti-CD38 antibodies (daratumumab, isatuximab) incorporated into frontline therapy have consistently increased rates of very good partial response (VGPR), complete response (CR), and MRD negativity when added to PI-IMiD doublets/triplets [36,37].

A major practice evolution has been the move from triplets to quadruplet induction. Randomized and large phase 2/3 datasets (GRIFFIN, GMMG-HD7, and several contemporary trials and meta-analyses) demonstrate that quadruplets containing an anti-CD38 antibody plus a PI + IMiD + dexamethasone produce higher depth of response and improved MRD-negativity rates than historical triplets, translating into improved progression-free survival in multiple cohorts [38-40]. Importantly, quadruplet approaches require attention to sequencing (e.g., optimizing stem-cell collection after IMiD exposure), infection prophylaxis, and toxicity monitoring, but data from MASTER/GRIFFIN suggest that stem-cell mobilization remains feasible after modern quadruplet induction [41].

Role of Risk-Adapted Therapy

Risk-adapted approaches are increasingly formalized: comprehensive genomic/cytogenetic profiling (FISH and sequencing), baseline tumor burden, EMD, and MRD status inform escalation to intensified regimens (e.g., carfilzomib-containing quadruplets, tandem ASCT in selected centers) or de-escalation in very-low-risk patients [42]. Contemporary guidelines encourage tailoring induction intensity and post-remission consolidation to individual risk, with MRD used as an early decision pivot in many prospective frameworks.

Stem cell transplantation and beyond

Contemporary Transplant Strategies, Tandem SCT

**Figure 3:**
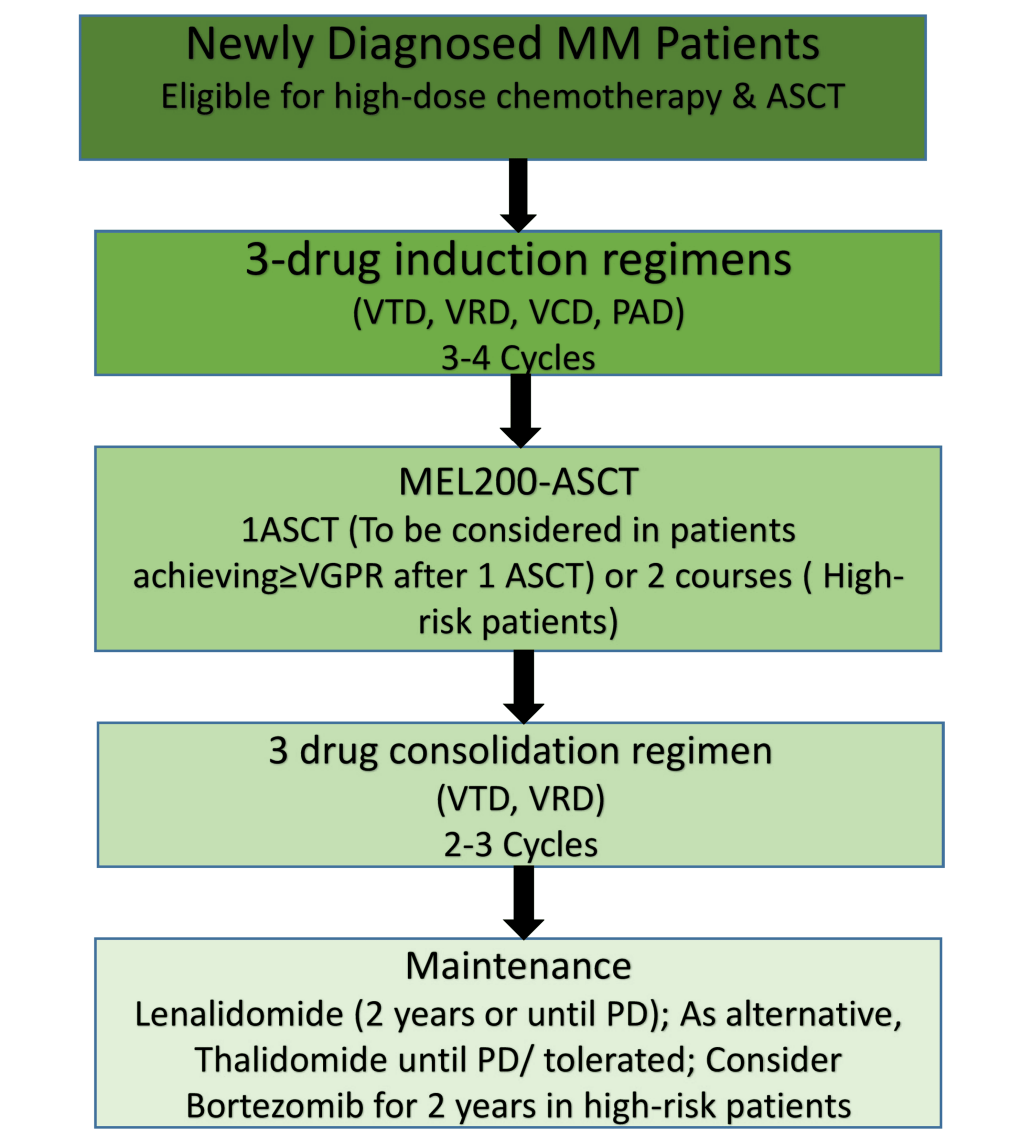
Treatment algorithm for newly diagnosed multiple myeloma (MM) patients eligible for autologous stem cell transplantation (ASCT) This flowchart summarizes the standard management pathway for transplant-eligible MM patients. Induction therapy typically consists of three-drug regimens such as bortezomib-thalidomide-dexamethasone (VTD), bortezomib-lenalidomide-dexamethasone (VRD), bortezomib-cyclophosphamide-dexamethasone (VCD), or bortezomib-doxorubicin-dexamethasone (PAD) for three to four cycles. High-dose melphalan (MEL200) followed by ASCT is performed, with consideration of a second ASCT in high-risk patients or those with suboptimal response (<VGPR). Post-transplant consolidation with two to three cycles of triplet therapy (e.g., VTD, VRD) may be given, followed by maintenance. Maintenance options include lenalidomide for two years or until progression, thalidomide if tolerated, and bortezomib for two years in high-risk patients [42]. Abbreviations: PD, progressive disease; VGPR, very good partial response Image credit: Nejma Azeez

Autologous stem cell transplantation (ASCT) remains a standard for eligible NDMM patients and continues to provide a durable PFS benefit even in the era of powerful induction regimens; however, the role of tandem ASCT is now more selective. Recent registry and randomized evaluations suggest limited incremental benefit of routine tandem ASCT in the maintenance era, though it may still be considered for selected high-risk patients who fail to achieve adequate depth of response after the first ASCT [43]. Several groups are testing strategies combining intensive quadruplet induction with either single ASCT, deferred transplant, or tandem ASCT based on MRD and cytogenetic risk. The standard therapeutic pathway for transplant-eligible patients involves triplet induction therapy, high-dose melphalan with ASCT, followed by consolidation and maintenance, tailored to disease risk and treatment response (Figure 3).

Maintenance Therapies and Novel Oral Regimens

Post-transplant maintenance remains a challenge to prolong remission. Lenalidomide maintenance after ASCT continues to offer PFS and OS advantage for standard-risk disease; for high-risk patients, combinations (e.g., bortezomib-based or anti-CD38 plus IMiD maintenance) are being explored to improve outcomes [41,44]. Recent trials indicate that anti-CD38 maintenance (daratumumab + lenalidomide) increases rates of MRD conversion and PFS vs. lenalidomide alone, albeit with increased cytopenias and infection risk that must be managed [44]. Simultaneously, convenience and adherence have improved with the evolution of oral options and targeted maintenance strategies (e.g., oral CELMoDs, selective pathways), expanding outpatient, long-term disease control possibilities.

Novel immunotherapies

CAR-T Cell Therapy: Product Evolution, Real-World Data, Challenges

CAR-T cell therapy has transformed the treatment of relapsed/refractory multiple myeloma (RRMM). Products, such as idecabtagene vicleucel (ide-cel) and ciltacabtagene autoleucel (cilta-cel), demonstrate overall response rates (ORR) of 70-90% in heavily pretreated patients, with significant rates of MRD negativity [45,46]. Real-world data confirm efficacy but highlight variability in adverse events, including cytokine release syndrome (CRS) and neurotoxicity [47]. Emerging strategies, including dual-target CAR-T constructs, aim to mitigate antigen escape and enhance durability of responses [48].

Bispecific Antibodies and Antibody-Drug Conjugates (ADCs)

BsAbs, such as teclistamab and talquetamab, recruit T cells to malignant plasma cells, offering off-the-shelf immunotherapy with ORRs of 60-70% in RRMM [49,50]. BsAbs present manageable toxicity profiles and do not require personalized manufacturing. ADCs like belantamab mafodotin target BCMA-positive myeloma cells while delivering cytotoxic payloads; ocular toxicity remains a key adverse effect, requiring monitoring and dose modification [51].

Vaccines and Immune Checkpoint Inhibitors: Emerging Frontiers

Tumor-specific vaccines are under evaluation to prime adaptive immunity against myeloma cells, with early-phase trials demonstrating immunogenicity but limited clinical benefit to date [52]. Immune checkpoint inhibitors (e.g., PD-1/PD-L1 blockade) have shown modest efficacy as monotherapy; combination strategies with immunomodulatory drugs (IMiDs) or CAR-T are being explored to overcome immune evasion [53].

Management of non-myeloma PCDs

Waldenström’s Macroglobulinemia (WM): BTK and PI3K Inhibitors

WM is characterized by an IgM monoclonal protein and lymphoplasmacytic infiltration. Bruton tyrosine kinase (BTK) inhibitors, including ibrutinib and zanubrutinib, provide durable responses and are frontline therapies in symptomatic patients [54]. PI3K inhibitors target downstream survival pathways, particularly in MYD88-mutant disease, with ongoing studies evaluating efficacy and safety [55].

AL Amyloidosis: Daratumumab + CyBorD, Novel Fibril-Targeting Agents

AL amyloidosis results from misfolded light-chain deposition in organs. The combination of daratumumab with CyBorD (cyclophosphamide, bortezomib, dexamethasone) yields rapid hematologic and organ responses, becoming standard first-line therapy [56]. Novel fibril-targeting antibodies, such as CAEL-101, aim to remove deposited amyloid, showing promising early-phase results in improving cardiac and renal function [57].

Plasmacytomas and EMD

Plasmacytomas and EMD represent aggressive PCD manifestations. Management includes local radiotherapy for solitary lesions and systemic therapy for disseminated disease [58]. High-risk cytogenetics and EMD presence often necessitate incorporation of novel agents such as proteasome inhibitors, IMiDs, and anti-CD38 antibodies [59].

Special populations and supportive care: moving beyond survival

Elderly and Frail Patients: Geriatric-Adapted Scoring and Therapy De-escalation

MM predominantly affects older adults, necessitating tailored treatment approaches. The IMWG frailty score, incorporating age, functional status, comorbidities, and laboratory parameters, stratifies patients into fit, intermediate-fit, and frail categories [60,61]. This stratification guides therapy intensity, with frail patients often benefiting from de-escalated regimens to minimize toxicity and enhance QoL [60-62]. Studies have shown that frailty assessments can predict treatment-related toxicity and overall survival, emphasizing the need for individualized treatment plans [62].

Additionally, geriatric assessments, including evaluations of cognitive function, nutritional status, and social support, are crucial in managing elderly patients with MM [61-63]. These assessments help identify vulnerabilities and guide decisions regarding treatment intensity and supportive care. Implementing geriatric-adapted scoring systems has been associated with improved patient outcomes and reduced hospitalization rates [63].

Renal Impairment: Plasma Exchange and Novel Dialysis Filters

Renal impairment is a common complication in MM, often resulting from cast nephropathy or light chain deposition [64,65]. While plasma exchange has been utilized to remove FLCs, evidence supporting its efficacy is limited [64]. A study by Clark et al. found no conclusive evidence that plasma exchange substantially reduces death or dialysis dependence in patients with acute renal failure at the onset of MM [64].

Emerging therapies include high-cutoff hemodialysis membranes, which effectively remove excess light chains and may improve renal function in some patients [65,66]. These novel dialysis filters have shown promise in small studies, suggesting potential benefits in reversing renal impairment associated with MM [66].

Additionally, the use of anti-myeloma therapy alongside high-cutoff hemodialysis membranes has been explored as a strategy to potentially reverse renal impairment in dialysis patients [65-67]. However, further research is needed to establish the efficacy and safety of this combined approach [67].

Skeletal Disease: Denosumab vs. Bisphosphonates and Novel Bone-Targeted Agents

Bone disease is a common complication in MM, leading to skeletal-related events such as fractures and pain [68,69]. Bisphosphonates, such as zoledronic acid, have been standard in preventing these events [68]. Denosumab, a monoclonal antibody targeting RANKL, has shown superior efficacy in certain studies and is preferred in patients with renal impairment due to its non-reliance on renal clearance [68-70]. A meta-analysis demonstrated that denosumab was more effective than bisphosphonates in increasing bone mineral density in postmenopausal women previously treated with oral bisphosphonates [70].

Novel bone-targeted agents, such as osteoprotegerin analogs and sclerostin inhibitors, are under investigation for their potential to further mitigate bone-related complications in MM [71,72]. These agents aim to regulate bone metabolism pathways more precisely, offering hope for improved management of myeloma-induced bone disease [71,72].

Thromboprophylaxis and Infection Prevention

Patients with MM, particularly those receiving IMiDs, are at increased risk for venous thromboembolism (VTE) [73,74]. Risk assessment models like IMPEDE-VTE and SAVED help identify high-risk individuals [73,74]. Prophylactic strategies include low-molecular-weight heparin (LMWH), direct oral anticoagulants (DOACs), or aspirin, tailored to individual risk profiles [73-75]. The IMWG guidelines recommend primary VTE prophylaxis for all patients treated with IMiDs in combination with dexamethasone, in the absence of significant comorbidities [75].

Infection prevention is critical, especially during periods of neutropenia or following stem cell transplantation [76,77]. Prophylactic antibiotics, antifungals, and antiviral agents are commonly used, with adjustments based on local epidemiology and patient-specific factors [76-78]. Monitoring and prompt treatment of infections are essential to reduce morbidity and mortality in this patient population [77,78].

QoL, Survivorship, and Palliative Care

QoL considerations are paramount in MM management [79,80]. Patients often experience fatigue, pain, neuropathy, and psychological distress [79-81]. Early integration of palliative care has been shown to improve symptom management and patient satisfaction [79,80]. A study by Rüsing et al. highlighted that patients with MM and survivors experience substantive long-term QoL issues, underscoring the need for comprehensive symptom management, integrated palliative care, and enhancement of social and emotional support [82].

Survivorship care plans should address long-term complications, including secondary malignancies, cardiovascular health, and psychosocial support [82,83]. The International Myeloma Foundation's Nurse Leadership Board has developed a survivorship care plan that emphasizes the identification of unmet needs and the importance of prompt intervention to improve QoL and overall survival [83,84].

Despite improvements in treatment options, providing optimal care to patients with MM at the end of life remains an unmet need [84]. Patients with MM typically have high rates of emergency department visits and hospitalizations at the end of life, indicating the necessity for better palliative care integration [84,85]. Table 2 outlines novel therapeutic strategies in MM, including response rates, toxicity profiles, and their current clinical status.

**Table 2:** Novel therapeutic strategies and their outcomes in multiple myeloma Abbreviations: ADCs, antibody-drug conjugates; CAR-T, chimeric antigen receptor-T; CELMoDs, cereblon E3 ligase modulators; Dara-VRd, daratumumab + bortezomib + lenalidomide + dexamethasone; Isa-KRd, isatuximab + carfilzomib + lenalidomide + dexamethasone; mAbs, monoclonal antibodies; MRD, minimal residual disease; ORR, overall response rate; PFS, progression-free survival; RRMM, relapsed or refractory multiple myeloma; TBD, to be determined

Therapy	Example Agents	Response Rates	Key Toxicities	Current Status	Reference
CAR-T Therapy	Ide-cel, Cilta-cel	ORR 70-90%, MRD-negativity high	CRS, neurotoxicity	Approved for RRMM, trials in frontline	[45-48]
Bispecific Antibodies	Teclistamab, Talquetamab	ORR 60-70%	Cytopenias, infections	Approved for RRMM	[49-50]
ADCs	Belantamab mafodotin	ORR 30-40%	Ocular toxicity	Conditional approval, ongoing studies	[51]
Quadruplet Induction	Dara-VRd, Isa-KRd	High MRD-negativity, improved PFS	Myelosuppression, infections	Frontline standard in many centers	[38-40]
Maintenance	Lenalidomide, Dara+Len	Improved OS/PFS	Cytopenia, infections	Standard of care	[41,44]
Novel Agents	CELMoDs, fibril-targeting mAbs, vaccines	Early promise	TBD	Under investigation	[56-57,71-72]

Unmet challenges and future directions

Drug Resistance and Clonal Escape

MM is characterized by genetic heterogeneity and clonal evolution, leading to therapy resistance and disease relapse [86-87]. Mechanisms, such as antigen loss (e.g., BCMA or GPRC5D), epigenetic changes, and immune evasion, contribute to clonal escape and resistance to treatments like CAR-T and BsAb [87-88]. Recent studies have identified therapy-induced clonal evolution as a significant factor hindering long-term remission [88-89]. Understanding these mechanisms is crucial for developing strategies to overcome resistance and achieve durable responses [89].

Cost and Accessibility of Advanced Therapies

The high cost of MM therapies poses a significant barrier to access, particularly in low- and middle-income countries (LMICs) [90-91]. For instance, the cost of anti-CD38 monoclonal antibodies in combination with other agents can exceed $300,000 per year [90]. A survey of oncologists revealed that while most MM therapies are accessible in high-income countries, access is limited in LMICs due to financial constraints and insufficient regulatory approvals [91-92]. Efforts to reduce costs and improve accessibility are essential to ensure equitable treatment for all MM patients [92].

Need for Global Collaborative Trials (LMIC Focus)

Conducting clinical trials in LMICs is challenging due to factors like limited infrastructure, regulatory hurdles, and financial constraints [91,93]. A systematic review found that high-income countries enrolled patients in 100% of trials resulting in FDA approvals, while seven countries in Central and Eastern Europe had no access to MM clinical trials [93]. Collaborations between high-income and LMICs are crucial to develop global health approaches that benefit these regions [91,93]. Such partnerships can enhance research capacity and improve patient outcomes worldwide [93].

Integration of Multi-omics + AI-Driven Personalized Algorithms

Advancements in multi-omics technologies and AI are revolutionizing MM treatment by enabling personalized medicine [94-95]. The Myeloma VAE (MyeVAE) model integrates genomic, transcriptomic, and clinical data to predict patient outcomes and guide therapy decisions [94]. Machine learning approaches utilizing proteomics data have also been employed to identify personalized treatments in MM patients [95-96]. These innovations hold promise for optimizing treatment strategies and improving patient outcomes [96].

The “Cure vs. Control” Debate in Myeloma

The debate between aiming for a cure vs. disease control in MM treatment reflects differing philosophies in patient management [97-98]. While some advocate for aggressive treatment strategies to achieve long-term remission or cure, others emphasize the importance of controlling the disease to maintain QoL [97]. Recent advancements, such as the CARTITUDE-1 trial, have shown promising results in achieving deep and durable responses, suggesting that a cure may be achievable in a subset of patients [99]. However, the optimal approach remains a subject of ongoing research and discussion.

## Conclusions

The diagnosis and management of PCDs are evolving rapidly, with important implications for everyday clinical practice. Advanced diagnostic tools, including MS, ultrasensitive sFLC assays, NGF or sequencing for MRD, and sensitive whole-body imaging, now enable earlier detection, improved risk stratification, and more accurate assessment of treatment response, particularly in patients with low-level or suspected residual disease.

Therapeutically, immunotherapy-based strategies such as anti-CD38 monoclonal antibodies, bispecific T-cell engagers, and CAR-T cell therapies have shifted treatment paradigms toward deeper and more durable responses across disease stages. Increasing integration of MRD status and biological risk factors is supporting individualized, response-adapted treatment decisions. Moving forward, clinicians will need to balance treatment intensity with toxicity, cost, and patient-centered outcomes, reinforcing a precision-guided and personalized approach to PCD care.

## References

[1] Awada H, Thapa B, Awada H, Dong J, Gurnari C, Hari P, Dhakal B (2021). A comprehensive review of the genomics of multiple myeloma: evolutionary trajectories, gene expression profiling, and emerging therapeutics. Cells.

[2] Hanamura I, Karnan S, Ota A, Takami A (2025). Primary plasma cell leukemia: recent advances in molecular understanding and treatment approaches. Int J Mol Sci.

[3] Diao X, Ben T, Cheng S, Niu S, Gao L, Xia N (2025). Global, regional, and national multiple myeloma burden from 1990 to 2021: a systematic analysis for of the Global Burden of Disease Study 2021. BMC Public Health.

[4] Choon-Quinones M, Zelei T, Németh B (2023). Systematic literature review of health economic models developed for multiple myeloma to support future analyses. J Med Econ.

[5] Cook R (2008). Economic and clinical impact of multiple myeloma to managed care. J Manag Care Pharm.

[6] Dhakal B, Girnius S, Hari P (2016). Recent advances in understanding multiple myeloma. F1000Res.

[7] Jamet B, Carlier T, Bailly C (2023). Hybrid simultaneous whole-body 2-[(18)F]FDG-PET/MRI imaging in newly diagnosed multiple myeloma: first diagnostic performance and clinical added value results. Eur Radiol.

[8] Gauthaman DK, Muthukrishnan I, Acharya KA, Simon S (2025). Ga-68 Pentixafor PET/CT in multiple myeloma and its correlation with clinical parameters: institutional pilot study. Ann Nucl Med.

[9] Navab R, Futela P, Kumari V, Valecha J, Gupta RB, Jain R (2025). Advancing multiple myeloma immunotherapy: a review of chimeric antigen receptor T-cell and bispecific T-cell engagers cell therapies in revolutionizing treatment. Iran J Med Sci.

[10] Lee H, Neri P, Bahlis NJ (2023). Current use of bispecific antibodies to treat multiple myeloma. Hematology Am Soc Hematol Educ Program.

[11] Dasari S, Kohlhagen MC, Dispenzieri A (2022). Detection of plasma cell disorders by mass spectrometry: a comprehensive review of 19,523 cases. Mayo Clin Proc.

[12] Murray DL, Puig N, Kristinsson S (2021). Mass spectrometry for the evaluation of monoclonal proteins in multiple myeloma and related disorders: an International Myeloma Working Group Mass Spectrometry Committee Report. Blood Cancer J.

[13] Liyasova M, McDonald Z, Taylor P (2021). A personalized mass spectrometry-based assay to monitor m-protein in patients with multiple myeloma (EasyM). Clin Cancer Res.

[14] Treger RS, Mathias PC, Cowan AJ (2024). Data analytics improves the diagnostic accuracy of serum free light chain results for detecting monoclonal gammopathy. Am J Clin Pathol.

[15] Sandfeld-Paulsen B, Aggerholm-Pedersen N, Samson MH, Møller HJ (2022). A cohort study of free light chain ratio in combination with serum protein electrophoresis as a first-line test in general practice. Cancers (Basel).

[16] Askari E, Dispenzieri A, Buadi FK (2025). Value of serum-free light chain measurements in response and progression assessment in multiple myeloma with monoclonal protein measurable by electrophoresis. Blood Cancer J.

[17] Tacchetti P, Pezzi A, Zamagni E (2017). Role of serum free light chain assay in the detection of early relapse and prediction of prognosis after relapse in multiple myeloma patients treated upfront with novel agents. Haematologica.

[18] Jevremovic D, Shi M, Horna P (2024). Real-life sensitivity of flow cytometry minimal residual disease assessment for plasma cell neoplasms. Blood Cancer J.

[19] Flores-Montero J, Sanoja-Flores L, Paiva B (2017). Next generation flow for highly sensitive and standardized detection of minimal residual disease in multiple myeloma. Leukemia.

[20] Oliva S, Genuardi E, Paris L (2023). Prospective evaluation of minimal residual disease in the phase II FORTE trial: a head-to-head comparison between multiparameter flow cytometry and next-generation sequencing. EClinicalMedicine.

[21] Hillengass J, Usmani S, Rajkumar SV (2019). International Myeloma Working Group consensus recommendations on imaging in monoclonal plasma cell disorders. Lancet Oncol.

[22] Lapa C, Schreder M, Schirbel A (2017). [(68)Ga]Pentixafor-PET/CT for imaging of chemokine receptor CXCR4 expression in multiple myeloma - comparison to [(18)F]FDG and laboratory values. Theranostics.

[23] Messiou C, Hillengass J, Delorme S (2019). Guidelines for acquisition, interpretation, and reporting of whole-body MRI in myeloma: myeloma response assessment and diagnosis system (MY-RADS). Radiology.

[24] Sachpekidis C, Enqvist O, Ulén J (2024). Artificial intelligence-based, volumetric assessment of the bone marrow metabolic activity in [(18)F]FDG PET/CT predicts survival in multiple myeloma. Eur J Nucl Med Mol Imaging.

[25] Hussain Z, De Brouwer E, Boiarsky R (2024). Joint AI-driven event prediction and longitudinal modeling in newly diagnosed and relapsed multiple myeloma. NPJ Digit Med.

[26] Kehl N, Kilian M, Michel J (2022). IgE type multiple myeloma exhibits hypermutated phenotype and tumor reactive T cells. J Immunother Cancer.

[27] Nafría Jiménez B, Oliveros Conejero R (2022). IgE multiple myeloma: detection and follow-up. Adv Lab Med.

[28] Narayanan G, Thambi SM, Prabhakaran PK (2023). Biclonal gammopathy - a single-center experience. Iraqi J Hematol.

[29] Giordano L, Cacciola R, Barone P (2024). Autoimmune diseases and plasma cell dyscrasias: pathogenetic, molecular and prognostic correlations. Diagnostics (Basel).

[30] Schoot TS, van Apeldoorn M, Sinnige HA, Beutler JJ (2020). Monoclonal gammopathy with significance: case series and literature review. Neth J Med.

[31] Buxbaum JN, Eisenberg DS, Fändrich M (2024). Amyloid nomenclature 2024: update, novel proteins, and recommendations by the International Society of Amyloidosis (ISA) Nomenclature Committee. Amyloid.

[32] Lee K, Kourelis T, Tschautscher M (2025). Capillary leak phenotype as a major cause of death in patients with POEMS syndrome. Leukemia.

[33] Shi QL, Xu Y, Wang J, Jin YY, Zhang R, Li JY, Chen LJ (2024). The therapeutic effect and prognostic value of oligoclonal bands after autologous stem cell transplant in patients with multiple myeloma. Zhonghua Yi Xue Za Zhi.

[34] Fernández de Larrea C, Tovar N, Cibeira MT (2011). Emergence of oligoclonal bands in patients with multiple myeloma in complete remission after induction chemotherapy: association with the use of novel agents. Haematologica.

[35] Georgiopoulos G, Makris N, Laina A (2023). Cardiovascular toxicity of proteasome inhibitors: underlying mechanisms and management strategies: JACC: CardioOncology state-of-the-art review. JACC CardioOncol.

[36] Ye L, Zhou F, Cheng D (2023). Efficacy and safety of anti-CD38 monoclonal antibodies in patients with relapsed/refractory multiple myeloma: a systematic review and meta-analysis with trial sequential analysis of randomized controlled trials. Front Oncol.

[37] Goldschmidt H, Mai EK, Bertsch U (2022). Addition of isatuximab to lenalidomide, bortezomib, and dexamethasone as induction therapy for newly diagnosed, transplantation-eligible patients with multiple myeloma (GMMG-HD7): part 1 of an open-label, multicentre, randomised, active-controlled, phase 3 trial. Lancet Haematol.

[38] Voorhees PM, Kaufman JL, Laubach J (2020). Daratumumab, lenalidomide, bortezomib, and dexamethasone for transplant-eligible newly diagnosed multiple myeloma: the GRIFFIN trial. Blood.

[39] Joseph NS, Kaufman JL, Gupta VA (2024). Quadruplet therapy for newly diagnosed myeloma: comparative analysis of sequential cohorts with triplet therapy lenalidomide, bortezomib and dexamethasone (RVd) versus daratumamab with RVD (DRVd) in transplant-eligible patients. Blood Cancer J.

[40] Kaiser MF, Hall A, Walker K (2023). Daratumumab, cyclophosphamide, bortezomib, lenalidomide, and dexamethasone as induction and extended consolidation improves outcome in ultra-high-risk multiple myeloma. J Clin Oncol.

[41] Chhabra S, Callander N, Watts NL (2023). Stem cell mobilization yields with daratumumab- and lenalidomide-containing quadruplet induction therapy in newly diagnosed multiple myeloma: findings from the MASTER and GRIFFIN trials. Transplant Cell Ther.

[42] Rajkumar SV (2024). Multiple myeloma: 2024 update on diagnosis, risk-stratification, and management. Am J Hematol.

[43] Venner CP, Duggan P, Song K (2024). Tandem autologous stem cell transplantation does not benefit high-risk myeloma patients in the maintenance era: real-world results from the Canadian Myeloma Research Group database. Transplant Cell Ther.

[44] Badros A, Foster L, Anderson LD Jr (2025). Daratumumab with lenalidomide as maintenance after transplant in newly diagnosed multiple myeloma: the AURIGA study. Blood.

[45] Munshi NC, Anderson LD Jr, Shah N (2021). Idecabtagene vicleucel in relapsed and refractory multiple myeloma. N Engl J Med.

[46] Ri M, Suzuki K, Ishida T (2022). Ciltacabtagene autoleucel in patients with relapsed/refractory multiple myeloma: CARTITUDE-1 (phase 2) Japanese cohort. Cancer Sci.

[47] Mewawalla P, Dileo R, Yin Y (2025). Real-world outcomes of sequential BCMA-directed therapies in relapsed refractory multiple myeloma after belantamab exposure. Clin Lymphoma Myeloma Leuk.

[48] Simon S, Riddell SR (2020). Dual targeting with CAR T cells to limit antigen escape in multiple myeloma. Blood Cancer Discov.

[49] Ishida T, Kuroda Y, Matsue K (2025). A Phase 1/2 study of teclistamab, a humanized BCMA × CD3 bispecific Ab in Japanese patients with relapsed/refractory MM. Int J Hematol.

[50] Schinke C, Touzeau C, Oriol A (2025). Talquetamab improves patient-reported symptoms and health-related quality of life in relapsed or refractory multiple myeloma: results from the phase 1/2 MonumenTAL-1 study. Cancer.

[51] Lonial S, Lee HC, Badros A (2020). Belantamab mafodotin for relapsed or refractory multiple myeloma (DREAMM- 2): a two-arm, randomised, open-label, phase 2 study. Lancet Oncol.

[52] Martino EA, Vigna E, Bruzzese A (2025). Vaccination in multiple myeloma: challenges and strategies. Eur J Haematol.

[53] Liu Z, Xu X, Liu H, Zhao X, Yang C, Fu R (2023). Immune checkpoint inhibitors for multiple myeloma immunotherapy. Exp Hematol Oncol.

[54] Treon SP, Tripsas CK, Meid K (2015). Ibrutinib in previously treated Waldenström's macroglobulinemia. N Engl J Med.

[55] Yang J, Nie J, Ma X, Wei Y, Peng Y, Wei X (2019). Targeting PI3K in cancer: mechanisms and advances in clinical trials. Mol Cancer.

[56] Kastritis E, Palladini G, Minnema MC (2021). Daratumumab-based treatment for immunoglobulin light-chain amyloidosis. N Engl J Med.

[57] Edwards CV, Rao N, Bhutani D (2021). Phase 1a/b study of monoclonal antibody CAEL-101 (11-1F4) in patients with AL amyloidosis. Blood.

[58] Li Y, Sun Z, Qu X (2022). Advances in the treatment of extramedullary disease in multiple myeloma. Transl Oncol.

[59] Liang D, Yan Y, Wang Q (2025). Clinical outcome of extramedullary multiple myeloma in the era of novel agents: insights from a multicenter study. Hematol Oncol.

[60] Cook G, Pawlyn C, Cairns DA (2022). Defining FiTNEss for treatment for multiple myeloma. Lancet Healthy Longev.

[61] Murillo A, Cronin AM, Laubach JP (2019). Performance of the International Myeloma Working Group myeloma frailty score among patients 75 and older. J Geriatr Oncol.

[62] Wildes TM, Tuchman SA, Klepin HD (2019). Geriatric assessment in older adults with multiple myeloma. J Am Geriatr Soc.

[63] Manapuram S, Hashmi H (2018). Treatment of multiple myeloma in elderly patients: a review of literature and practice guidelines. Cureus.

[64] Clark WF, Stewart AK, Rock GA (2005). Plasma exchange when myeloma presents as acute renal failure: a randomized, controlled trial. Ann Intern Med.

[65] Xing Y, Yan J, Yu Z (2022). High-cutoff hemodialysis in multiple myeloma patients with acute kidney injury. Front Oncol.

[66] Favà A, Fulladosa X, Montero N, Draibe J, Torras J, Gomà M, Cruzado JM (2018). Treatment of multiple myeloma with renal involvement: the nephrologist's view. Clin Kidney J.

[67] Latcha S, Leung N (2023). High-cutoff hemodialysis therapy for patients with light chain cast nephropathy and aki requiring dialysis: PRO. Kidney360.

[68] Goldstein DA (2018). Denosumab for bone lesions in multiple myeloma - what is its value?. Haematologica.

[69] Terpos E, Raje N, Croucher P (2021). Denosumab compared with zoledronic acid on PFS in multiple myeloma: exploratory results of an international phase 3 study. Blood Adv.

[70] Tanveer N, Hussein S, Pingili S (2023). Multiple myeloma and the role of bisphosphonates in its management. Cureus.

[71] Lin CH, Tariq MJ, Ullah F (2024). Current novel targeted therapeutic strategies in multiple myeloma. Int J Mol Sci.

[72] Ngomdir L, Bharathwaj VV, Nimmy P (2023). Therapeutic use of anti-sclerostin antibody in the treatment of multiple myeloma: a systematic review. J Pharm Bioallied Sci.

[73] Covut F, Sanfilippo KM (2022). Mitigating the risk of venous thromboembolism in patients with multiple myeloma receiving immunomodulatory-based therapy. Hematology Am Soc Hematol Educ Program.

[74] Frenzel L (2025). Thromboprophylaxis in multiple myeloma. Res Pract Thromb Haemost.

[75] De Stefano V, Larocca A, Carpenedo M (2022). Thrombosis in multiple myeloma: risk stratification, antithrombotic prophylaxis, and management of acute events. A consensus-based position paper from an ad hoc expert panel. Haematologica.

[76] Raje NS, Anaissie E, Kumar SK (2022). Consensus guidelines and recommendations for infection prevention in multiple myeloma: a report from the International Myeloma Working Group. Lancet Haematol.

[77] Miceli TS, Gonsalves WI, Buadi FK (2021). Supportive care in multiple myeloma: current practices and advances. Cancer Treat Res Commun.

[78] Blimark C, Holmberg E, Mellqvist UH (2015). Multiple myeloma and infections: a population-based study on 9253 multiple myeloma patients. Haematologica.

[79] Mathew A, Farooqui HH, Kumar L (2021). Quality of life assessment & out-of-pocket expenditure in multiple myeloma: an observational study. Indian J Med Res.

[80] Shah D, Sparano F, Luo C (2024). Patient-reported outcome domains in multiple myeloma randomized controlled trials and association with survival outcomes. Ann Hematol.

[81] Eilert N, Murphy NJ, Cummins H, Houlihan E, Krawczyk J (2024). A multidisciplinary group-based survivorship intervention for those living with multiple myeloma: a feasibility study. Pilot Feasibility Stud.

[82] Rüsing L, Brunbauer C, Michel CS (2025). Integrating palliative care into multiple myeloma management : optimizing quality of life across the disease continuum. Wien Klin Wochenschr.

[83] (2025). IMF Nurse Leadership Board. IMF Nurse Leadership Board (NLB) Survivorship Care Plan for Multiple Myeloma. International Myeloma Foundation. https://www.myeloma.org/resource-library/imf-nurse-leadership-board-nlb-survivorship-care-plan-multiple-myeloma.

[84] Odejide OO, Li L, Cronin AM, Murillo A, Richardson PG, Anderson KC, Abel GA (2018). Meaningful changes in end-of-life care among patients with myeloma. Haematologica.

[85] Dahal R, Abdal Khader AHS, Kasire SP (2024). MM-039 Unmet needs related to experiences of myeloma patients and their caregivers and potential solutions: a systemic literature review. Clin Lymphoma Myeloma Leuk.

[86] Forster S, Radpour R, Ochsenbein AF (2023). Molecular and immunological mechanisms of clonal evolution in multiple myeloma. Front Immunol.

[87] Marios P, Sungwoo A, Benjamin D (2024). Timing antigenic escape in multiple myeloma treated with t-cell redirecting immunotherapies. Blood.

[88] Cui J, Li X, Deng S (2024). Identification of therapy-induced clonal evolution and resistance pathways in minimal residual clones in multiple myeloma through single-cell sequencing. Clin Cancer Res.

[89] Botta C, Mendicino F, Martino EA (2021). Mechanisms of immune evasion in multiple myeloma: open questions and therapeutic opportunities. Cancers (Basel).

[90] Gupta-Werner N, Khare V, Macomson B, Medhekar R (2025). Cost of Anti-CD38 monoclonal antibodies in combination with bortezomib, lenalidomide and dexamethasone for the frontline treatment of transplant-ineligible patients with newly diagnosed multiple myeloma in the US. J Health Econ Outcomes Res.

[91] Atallah R, Shatnawi Y, Ayoobkhan MF (2025). Global access to multiple myeloma therapies. JCO Glob Oncol.

[92] Park Y, Kim S, Kim N (2025). Different level and difficulties with financial burden in multiple myeloma patients and caregivers: a dyadic qualitative study. Semin Oncol Nurs.

[93] Fatoki RA, Koehn K, Kelkar A (2022). Global myeloma trial participation and drug access in the era of novel therapies. JCO Glob Oncol.

[94] Kosvyra Α, Karadimitris Α, Papaioannou Μ, Chouvarda I (2024). Machine learning and integrative multi-omics network analysis for survival prediction in acute myeloid leukemia. Comput Biol Med.

[95] Katsenou A, O'Farrell R, Dowling P, Heckman CA, O'Gorman P, Bazou D (2023). Using proteomics data to identify personalized treatments in multiple myeloma: a machine learning approach. Int J Mol Sci.

[96] Alipour E, Pooyan A, Shomal Zadeh F, Darbandi AD, Bonaffini PA, Chalian M (2023). Current status and future of artificial intelligence in MM imaging: a systematic review. Diagnostics (Basel).

[97] Rajkumar SV (2008). Treatment of myeloma: cure vs control. Mayo Clin Proc.

[98] Bar N, Firestone RS, Usmani SZ (2023). Aiming for the cure in myeloma: putting our best foot forward. Blood Rev.

[99] Berdeja JG, Cohen AD, Martin T, Madduri D, Pacaud L, Jagannath S (2023). Plain language summary of the CARTITUDE-1 study of ciltacabtagene autoleucel for the treatment of people with relapsed or refractory multiple myeloma. Future Oncol.

